# The Impact of Early Rehabilitation and the Acceptance of the Disease on the Quality of Life of Patients after Hip Arthroplasty: An Observational Study

**DOI:** 10.3390/jcm13102902

**Published:** 2024-05-14

**Authors:** Joanna Chojnowska, Jolanta Lewko, Joanna Chilińska, Mateusz Cybulski, Wioletta Pogroszewska, Elżbieta Krajewska-Kułak, Regina Sierżantowicz

**Affiliations:** 1 Department of Physiotherapy, Faculty of Health Sciences, Academy of Lomza, 18-400 Lomza, Poland; jchojnowska@al.edu.pl (J.C.); miriam-77@wp.pl (W.P.); 2 Department of Primary Health Care, Medical University of Bialystok, 15-089 Bialystok, Poland; 3 Department of Nursing, Faculty of Health Sciences, Academy of Lomza,18-400 Lomza, Poland; jchilinska@al.edu.pl; 4 Department of Integrated Medical Care, Medical University of Bialystok, 15-089 Bialystok, Poland; mateusz.cybulski@umb.edu.pl (M.C.); elzbieta.krajewska@wp.pl (E.K.-K.); 5 Department of Surgical Nursing, Medical University of Bialystok, 15-089 Bialystok, Poland; renatasierz@wp.pl

**Keywords:** osteoarthritis of the hip joint, hip arthroplasty, rehabilitation, quality of life

## Abstract

**Background**: The early introduction of physiotherapy significantly shortens the time required for a patient to regain full mobility after hip arthroplasty. Obtaining the expected result is determined by cooperation with a physiotherapist and the patient’s involvement in the rehabilitation process. The aim of this study was to assess the quality of life, life satisfaction, and motor ability of patients after hip arthroplasty. **Methods**: This study included 147 patients who underwent hip arthroplasty at the Orthopedic and Trauma Department. The research material was collected using questionnaires, and the study used the Barthel Index (BI), Harris Hip Score (HHS), Visual Analogue Scale (VAS), Acceptance of Illness Scale (AIS), and Quality of Life Assessment Questionnaire (WHOQOL-BREF). In the studied group of patients, the Excia cementless endoprosthesis was primarily used (69.39%), as well as the Metha Short Hip prosthesis (15.65%), AM hip prosthesis (10.20%), and bipolar hip prosthesis (4.76%). **Results**: The analyzed group of patients included 95 women (64.63%) and 52 men (35.37%); the average age was 67 years. Six weeks after hip arthroplasty, mild disability occurred in 53.74% of the patients, while the remaining 46.26% had no disability, and 95.24% of the patients achieved a high level of acceptance of the disease and quality of life. **Conclusions**: Early improvement after hip replacement surgery contributes to eliminating the disability of the hip joint in the following areas: pain sensation, functionality, lack of deformation, and range of motion. Each subsequent stage of treatment increased the level of acceptance of the disease in the study group. The applied surgical treatment increased satisfaction with overall health and quality of life.

## 1. Introduction

Degenerative changes in the hip joints (coxarthrosis) are one of the most serious diseases affecting the musculoskeletal system which, with further development, lead to the complete destruction of these joints and result in surgical replacement of the joint. The scale of the problem is demonstrated by data from the Central Database of Arthroplasty of the National Health Fund in Poland, which show that, in the years 2005–2020, as many as 942.513 joint arthroplasty services were performed in Poland [1]. During this period, the highest growth dynamic was observed in the case of arthroplasty of the following joints: knee, elbow, and shoulder joints; however, in absolute values, the greatest increase was recorded for hip arthroplasty. During the 16-year period, the number of hip replacements increased from 26.092 in 2005 to 59.684 in 2019 and 46.258 in 2020 [1].

Postoperative physiotherapy should begin as soon as the patient returns from the operating room [2]. The main goals at this stage should be preventing dislocation of the prosthesis, preventing the development of pressure sores, preventing thromboembolic complications, implementing exercises for the operated limb, monitoring compliance with the introduced restrictions, and lifting the patient up to a semi-sitting position in bed [3]. The patient should be instructed on the limitations of movements in the hip joint; in addition, the validity and purposefulness of the rehabilitation should be explained [4].

Rehabilitation after surgery begins on the second day of hospitalization and lasts until the patient is discharged from the hospital. It includes free active exercises and decompression exercises in order to relieve the operated joint. The aim of these is to maintain the proper muscle strength and normalize the balance of soft tissue tension around the joint. Then, gait training begins and it is determined whether orthopedic equipment, such as a walker or crutches, is required. The type of equipment is determined by the physiotherapist, taking into account the age and condition of the patient [2].

As part of gait training, walking skills on flat terrain and on stairs are refined. When moving up the stairs, the order of each step is important. When beginning to climb the stairs, the non-operated limb is placed first, followed by the operated limb and crutches. When descending, the operated limb takes the first step together with the crutch, and then the non-operated limb is added [5]. Improving these skills continues until the patient leaves the department. In order to verify the achieved effects, gait tests are introduced, and the patient is subjected to them at the beginning and end of the recovery plan [6,7].

While patient rehabilitation is carried out, attention should be paid to important aspects of gait, i.e., the pelvic drop when patients place weight on the operated limb, lateralization of the trunk to the operated side, difficulties in maintaining balance, asymmetric weight placed on the limbs, shortening of the support phase of the limb after the procedure, and reduced pelvic rotation [6]. When carrying out rehabilitation in a hospital setting, the focus should also be on improving the patient’s self-service activities (i.e., sitting down/getting up from a chair, bed, or toilet; bed training mobility; and position changes), as well as everyday activities, taking into account the limitations resulting from movements prohibited after arthroplasty [8]. Indications to discontinue these exercises during the rehabilitation process include thrombophlebitis, dislocation of the endoprosthesis, severe pain in the thigh and pelvis, and acute circulatory and respiratory failure [9].

The aim of this study was to assess the impact of early rehabilitation and the acceptance of the disease on the quality of life of patients after hip arthroplasty.

## 2. Method

The study included 147 patients who underwent hip arthroplasty at the Orthopedic and Trauma Department of the Provincial Hospital in Lomża. The reason for the hospitalization of the patients was the diagnosis of osteoarthritis of the hip joint (77.55%) or the diagnosis of a hip fracture (22.45%).

The study included consecutive patients aged > 18 years, without COVID-19 infection, who were able to provide informed consent and did not meet the exclusion criteria. The criteria for excluding a patient from the study were locomotor or physical disability, mental illness, coagulation disorders, systemic disease requiring special perioperative care (e.g., intensive therapy, dialysis), or body mass index (BMI) > 40 kg/m^2^. In the studied group of patients, the Excia cementless endoprosthesis was primarily used (69.39%), as well as the Metha pedicle prosthesis (15.65%), AM hip prosthesis (10.20%), and bipolar hip prosthesis (4.76%).

The study was prospective, and the research material was collected using standardized tools such as the Barthel Index (BI), Harris Hip Score Scale (HHS), Visual Analogue Scale (VAS), Acceptance of Illness Scale (AIS), and Quality of Life Assessment Questionnaire (WHOQOL-BREF). Respondents completed questionnaires at three subsequent stages of treatment: before the surgery, on the 12th day after surgery, and 6 weeks after surgery.

### 2.1. Measurement

#### 2.1.1. Barthel Index (BI)

The BI is a tool for the clinical assessment of the ability to meet basic life needs and the degree of dependence on other people. The Barthel scale assesses 10 areas, including eating meals, moving around, maintaining personal hygiene, using the toilet, washing, bathing the entire body, moving on flat surfaces, going up and down the stairs, dressing and undressing, and controlling stool and urine.

The result of the scale is the sum of the points: ≤20, very serious disability; 25–45 points, serious disability; 50–70 points, average severity of disability; 75–95 points, mild disability; 100 points, full mobility [10].

#### 2.1.2. Acceptance of Illness Scale (AIS)

The AIS by Felton, Revenson, and Hinrichsen, with a Polish adaptation by Juczyński [11], is used to measure the degree of acceptance of the disease. It contains eight statements describing the negative consequences of poor health and can be applied to any disease.

In each statement, the respondent indicates their current state on a five-point scale, from 1 (“I strongly agree”) to 5 (“I strongly disagree”). The general measure of the degree of acceptance of the disease is the sum of the points, and its range is from 8 to 40 points. To determine the level of acceptance, three groups were created as follows: 8–18 points indicate no acceptance of the disease, 19–29 points indicate average acceptance, and 30–40 points indicate good acceptance.

The area of this study using the AIS evaluated the following: problems with adapting to limitations imposed by the disease, assessment of one’s own capabilities in the presence of the disease, one’s own feelings, and dependence on other people due to the disease [11]. A high score indicates acceptance of one’s own disease state and is manifested by the lack of negative emotions. Conversely, a low score indicates a lack of acceptance and adaptation to the disease and a strong sense of mental discomfort.

#### 2.1.3. WHOQOL-BREF Quality of Life Questionnaire

The WHOQOL-BREF is a shortened version of the quality-of-life survey. It was developed by the international Quality of Life Research Team at the World Health Organization with the intention of creating a universal research tool to measure quality of life regardless of cultural differences. It was created on the basis of the WHOQOL-100. It was adapted to Polish conditions by Wołowicka and Jaracz [12]. The questionnaire is a research tool intended to assess the quality of life of healthy and sick people, both for cognitive and clinical purposes. The WHOQOL-BREF contains 26 questions, and it allows one to determine quality of life in four areas: physical, mental, social, and environmental functioning. In terms of the scoring, the higher the number of points, the better the quality of life. In each area, the arithmetic mean is calculated and then multiplied by four to obtain results comparable to the WHOQOL-100 [12].

#### 2.1.4. Harris Hip Score (HHS)

The HHS was developed to evaluate the outcomes of hip surgery and is intended to evaluate various types of hip disabilities and treatments in the adult population. The pain domain includes the type of treatment, frequency of occurrence, and use of painkillers. The domain of function consists of an assessment of daily activities (using stairs, using public transport, walking, sitting, and self-care-related actions such as putting on socks or shoes). The range of motion domain measures hip flexion, hyperextension, abduction, external and internal rotation, and adduction. The deformation domain includes no hip flexion contracture, adduction contracture, internal rotation contracture, and shortening of limb length. The final domain defines the main disorders caused by pain. A maximum of 100 points can be obtained. The better the patient’s functioning, the better the result [13].

#### 2.1.5. Visual Analogue Scale (VAS)

The analog, visual pain rating scale is a reliable tool for determining the intensity of pain. Periodic and repeated measurements of pain intensity using the VAS enable the assessment of the effectiveness of analgesic treatments. The scale is a straight horizontal line of fixed length, typically 10 cm. The ends are defined as the extreme limits of the parameter, placed from the left (good) to the right (the worst). The patient indicates the intensity of pain (from 0, indicating no pain at all, to 10, the strongest pain imaginable) with a finger or a slider [13].

## 3. Statistical Analyses

The statistical analysis was performed using STATISTICA version 7.0 software from StatSoft Polska. Due to a non-normal distribution of the variables, the data analysis was performed using the non-parametric Kruskal–Wallis test to compare the distribution of a numerical feature in several groups, and the Mann–Whitney *U* test was used to assess differences in the distribution of the numerical feature in the two groups. ANOVA testing was used to assess the relationship between variables. Confidence intervals (CIs) were calculated for mean values. The analysis of the relationship between two numerical (ordinal) features consisted of determining the value of Spearman’s rank correlation coefficient (R) and performing a test of the statistical significance of the relationship under study. Spearman’s rank correlation coefficient is used to examine the relationship between two numerical features and ranges from -1 to 1. The strength of the correlation is indicated by the absolute value of the coefficient and by the direction of the sign. The following objective scale regarding the strength of correlation was adopted in this study: |rs| < 0.3, no correlation; 0.3 ≤ |rs| < 0.5, weak correlation; 0.5 ≤ |rs| < 0.7, average correlation; 0.7 ≤ |rs| < 0.9, strong correlation; 0.9 ≤ |rs| ≤ 1, very strong correlation. As a result of the statistical test, differences were considered statistically significant when *p* < 0.05.

## 4. Results

### 4.1. Characteristics of the Study Group

The study group consisted of 147 patients who underwent hip arthroplasty. The reason for hospitalization was the diagnosis of degenerative disease of the hip joint (coxarthrosis, 77.55%) or the diagnosis of hip fracture (22.45%). The hospitalization time in the analyzed cases varied from 12 to 75 days. Most patients were hospitalized for 12 days (60.54%), while hospitalization lasted no longer than 14 days for 93.88% of the patients.

The analyzed group of patients included 95 women (64.63%) and 52 men (35.37%). The age of the patients varied: up to 50 years (7.48%), 51–65 years (35.37%), 66–80 years (42.86%), >80 years (14.29%). The average age was 67 years. The respondents were residents of rural areas (46.94%) and urban areas (53.06%). The respondents mostly had a vocational education (46.26%) or secondary education (50.34%); people with higher education constituted 3.40% of the total. The respondents were mostly married (70.75%); the rest were single, including widowers (25.85%), divorced (2.72%), and single patients (0.68%). The living situation of the respondents was varied: only with a spouse (46.26%), with a spouse and children (25.85%), only with children (12.45%), and alone (8.84%). Most of the surveyed patients assessed their social and living conditions as good (82.99%) or average (17.01%) (Table 1).

### 4.2. Assessment of the Pain Level According to the VAS Depending on the Stage of Treatment

According to the VAS, the surgical treatment effectively reduced the level of pain experienced by the patient compared to the preoperative period. The average results obtained in Stages I and II were higher than those obtained 6 weeks after the procedure (Table 2).

### 4.3. Assessment of Hip Joint Disability according to the HHS Scale

According to the HHS scale, the treatment effectively increased the functional efficiency of the hip joint in the following areas: pain sensation, functionality, lack of deformation, and range of motion. The average results obtained in Stages I and II were lower than those obtained 6 weeks after the procedure (Table 3).

### 4.4. Assessment of the Patient’s Physical Impairment according to the Barthel Index

The patients’ independence improved with each stage of treatment: Stage I, 83.40 ± 23.60; Stage II, 90.54 ± 2.93; and Stage III, 96.97 ± 3.58. Cases of severe disability according to the Barthel Index occurred only in the first stage of the study and constituted 21.77% of the group; 57.82% had mild disability and 20.41% possessed full ability. At the second stage of the study, on the 12th day after the procedure, all patients were assessed as having mild disabilities. Six weeks after hip arthroplasty, mild disability occurred in 53.74% of the respondents while the remaining 46.26% had no disability (Table 4).

### 4.5. The Level of Acceptance of the Disease at Subsequent Stages of Treatment

It was observed that as the treatment progressed, the level of acceptance of the disease increased. At subsequent stages of treatment, the following mean results of the AIS were obtained: 28.68 ± 5.02, 29.81 ± 3.68, and 36.50 ± 2.01. In Stage III, according to the obtained median value, the AIS score for half of the examined patients was at least 37 points. A low level of acceptance of the disease was obtained only in the first stage of the pre-treatment examination in 8.84% of cases. The share of people with an average and high level of acceptance of the disease was comparable before and on the 12th day after the procedure. However, after the 6th week following the procedure, 95.24% of the respondents achieved a high level of acceptance of the disease according to the AIS, while the remaining 4.76% obtained an average result (Table 5).

### 4.6. Assessment of Quality of Life at Subsequent Stages of Treatment

At each stage of the study, patients rated their quality of life as the worst in the psychological area, followed by the environmental and social areas. At Stages I and II, the quality-of-life assessment obtained in the physical area was comparable to the other areas examined. However, at the third stage of the study, 6 weeks after the procedure, there was a significant increase in the quality of life in the physical field, where the average value was 16.36 ± 1.77, out of a possible maximum value of 20 points for each domain (Table 6).

### 4.7. The Impact of AIS Results on the WHOQOL-BREF Questionnaire

The highest quality-of-life scores were obtained in the group of patients with a high level of acceptance of the disease. At each stage of the study, acceptance of the disease influenced the assessment of quality of life. It was found that the higher the level of acceptance of the disease, the higher the patient’s quality-of-life assessment according to the WHOQOL-BREF (Table 7).

The highest health status scores were obtained in the group of patients with a high level of acceptance of the disease. At each stage of the study, acceptance of the disease influenced the assessment of health status. It was found that the higher the level of acceptance of the disease, the higher the patient’s assessment of their health status according to the WHOQOL-BREF.

The highest assessments of quality of life in the physical and the psychological fields were obtained in the group of patients with a high level of acceptance of the disease. At each stage of the study, acceptance of the disease influenced the assessment of quality of life in the physical and the psychological fields. It was found that the higher the level of acceptance of the disease, the higher the assessment of the quality of life in the physical and psychological fields according to the WHOQOL-BREF (Table 7).

The highest assessments of quality of life in the social field were obtained in the group of patients with a high level of acceptance of the disease. At each stage of the study, acceptance of the disease influenced the assessment of quality of life in the social field. It was found that the higher the level of acceptance of the disease, the higher the assessment of the quality of life in the social field according to the WHOQOL-BREF (Table 7).

The highest assessments of quality of life in the environmental field were obtained in the group of patients with a high level of acceptance of the disease. At each stage of the study, acceptance of the disease influenced the assessment of quality of life in the environmental field. It was found that the higher the level of acceptance of the disease, the higher the assessment of the quality of life in the environmental field according to the WHOQOL-BREF (Table 7).

### 4.8. Relationships between Psychometric Measures

At each stage of the study, the VAS results correlated significantly with the WHOQOL-BREF results. It was shown that the lower the level of pain experienced, the higher the assessment of the quality of health and life in all analyzed areas (Table 8).

Information about the correlation between various psychometric measures may be important from a practical point of view. If certain measures are highly correlated, it allows us to optimize the patient’s care process and achieve the highest possible quality of life, which is the priority of modern medicine.

Therefore, a correlation analysis was performed between the measures considered in the study by calculating the Spearman’s rank correlation coefficients. The analysis was carried out at the level of the three treatment stages examined: Stage I, before the procedure; Stage II, 12 days after the procedure; and Stage III, 6 weeks after the procedure.

The tables provide the values of the correlation coefficient, along with their statistical significance, assessed on the basis of the value of the test probability *p*-value, which, however, was not indicated in the table (due to the high complexity of the table, which could reduce the readability of the results). To facilitate the interpretation of the results, a statistically significant correlation at the *p* < 0.05 level was marked with the symbol *.

Table 9 shows the correlations between the psychometric measures at the first stage of the subjects’ treatment (before the treatment). Analyzing the results, it can be seen that there were significant correlations between all analyzed psychometric measures. In the period before the procedure, there were strong correlations between the level of hip joint disability in the areas of pain, functionality, lack of deformation, range of motion (HHS), and the assessment of the quality of health and life in all aspects (WHOQOL-BREF). It was also found that as the disability of the hip joint (HHS) increased, the level of pain experienced (VAS) increased, and the patient’s independence decreased (BI), while the level of acceptance of hip joint degeneration also decreased among the respondents. This means that at the first stage of the patient’s treatment (before the procedure), the most important factors were the shortest possible waiting time for a procedure, ensuring maximum comfort of living, and implementing individual pain therapy.

Table 9 shows the correlations between the psychometric measures at the second stage of the subjects’ treatment (12 days after the treatment). Analyzing the results, it can be concluded that there were significant correlations between the psychometric measures at this stage. Furthermore, 12 days after the procedure, the level of hip joint disability (HHS) significantly correlated with the assessment of the quality of health and life in all aspects (WHOQOL-BREF). However, no significant relationships were found between the level of hip joint disability (HHS) and the patient’s level of independence (Barthel). There was also no significant relationship between the patient’s level of independence (Barthel) and the assessment of the quality of health and life in the environmental field. It was found that the level of pain measured using the VAS correlated most strongly with the assessment of quality of life at this stage of treatment. This means that in the second stage of the patient’s treatment (12 days after the procedure), the patient did not focus on their functionality and independence, perceiving their limitations as normal at this point in the treatment, but it was important to provide the appropriate pain therapy, which influenced the patient’s assessment of their quality of life at this stage of the treatment.

Table 9 shows the correlations between the psychometric measures throughout the third stage of the subjects’ treatment (6 weeks after the treatment). Analyzing the results, it was found that there were significant correlations between all the analyzed psychometric measures. This corresponds to the observation made in the first stage of the study (before the procedure). It was found that the level of hip joint disability measured by the HHS scale had the greatest impact on the assessment of the quality of health and life. This means that during Stage III of the treatment, the patient’s satisfaction with health and life influences the recovery of hip joint function, which was also associated with a decrease in the level of pain felt (VAS). The psychometric measures mentioned above significantly correlated with the level of acceptance of the disease, which increased with improvement in the patient’s independence and functionality. This means that in the 6 weeks after the procedure, the patient waited to regain full mobility and to return to performing all social functions assigned to them. This effect mentioned was perceived as the expected therapeutic success of the treatment process. In the case of persistent limitations, it is recommended to educate the patient and their family regarding further treatment in the form of specialist treatment in a clinic, hospital, or sanatorium.

## 5. Discussion

Surgical treatment helps solve the most important problems related to the progression of joint diseases. The enormous pace of new technologies and design changes allow patients to return to the correct, more physiological biomechanics of the hip joint. The early introduction of physiotherapy significantly shortens the time needed to return to the fullest possible mobility. Obtaining the expected result in the patient is determined by cooperation with a physiotherapist and the patient’s involvement in the rehabilitation process [14].

Hip arthroplasty results in reduced disability of patients according to Szczepłek et al. [15]. The average HHS improved two-fold. Before the procedure, it was 37.07 points; after the procedure, it was 74.93 points. A positive correlation was confirmed between the age of the subjects and the HHS result, and hip arthroplasty significantly reduced the patients’ disability three months after the surgery [15]. In addition, Bodys-Cupak et al. [16] observed a clear decrease in disability in the group of “medium-severe” patients, from 31% before surgery to 7% after surgery. This was accompanied by a significant increase in the group of patients in the “light” condition, from 68% to 92%, according to the Kotel et al. [17], while the functional changes in the patients after hip arthroplasty were found to be very good and good. Stanek et al. [18] reported that, as a result of hip arthroplasty, a significant decrease was observed in the number of patients with limited walking ability; limited hip joint mobility; pain in the hip, buttock, and thigh; and leg contracture. The ability of patients to climb and walk up and down stairs significantly improved. This was the result of postoperative rehabilitation, which was used by 95% of the study group. In their study, Stanek et al. [18] showed that, as a result of implantation of a joint endoprosthesis, the degree of difficulty in performing all analyzed activities decreased significantly. This produced an increase in the independence of patients: the number of people requiring help from other people decreased from 37.5% to 20%. There was also an increase in satisfaction in the subjective assessment of one’s physical fitness. Leiss et al. [19], based on a 12-month observation, showed a significant improvement in the physical fitness and social functionality of people after hip arthroplasty. Larsen et al. [20] emphasized the importance of early rehabilitation after surgery for the therapeutic effect achieved. Kutzner et al. [21] found that a significant improvement in the fitness of patients after hip arthroplasty was noticeable in a group that received early rehabilitation in hospital conditions or the conventional method in home and outpatient conditions. The priority of modern medicine is to provide the highest possible quality of life by achieving the best possible health condition of a patient. According to the WHO definition, health is perceived in terms of physical, mental, and social well-being and not merely the absence of disease [12]. The aim of hip replacement is to eliminate the patient’s pain and disability, which leads to the recovery of physical, mental, and social functioning, resulting in an improvement in the quality of life in these aspects.

The study assessed the quality of life using the WHOQOL-BREF questionnaire. In the period before the surgery and after 12 days following the procedure, the quality-of-life assessment results were comparable, but they improved significantly after 6 weeks. Through analyzing the quality of life in individual WHOQOL-BREF domains, it was found that the quality of life in the mental, social, and environmental aspects was comparable, and the assessment in the physical area was significantly improved. It was shown that physical and functional fitness, as well as the relief of pain, confirmed by the results of the BI, HHS, and VAS, were important for improving the quality of life in the physical domain. In the analyzed group, the level of acceptance of the disease, assessed using the AIS, was also observed to increase with each subsequent stage of treatment.

The results of the conducted research regarding quality of life after hip arthroplasty are consistent with those of other authors. Research by Majda et al. [22] showed an improvement in the quality of life of patients in the following dimensions: life energy, pain, mobility, and in the sphere of life related to housework, free time management, social life, and hobbies. Similarly, Grochans et al. reported [23] a significant improvement in the quality of life of patients after the procedure, highlighting better mental health in women, improved physical functionality in older people, and alleviation of pain. Grochans et al. [23] emphasized that participation in rehabilitation before the procedure significantly improved the patient’s overall well-being after the procedure. However, Kania et al. [24] did not demonstrate an impact of preoperative rehabilitation on quality of life before surgery and the impact of postoperative rehabilitation on pain sensation. The authors noted an improvement in the subjects’ sleep quality and an increase in independence in everyday activities and climbing stairs. In addition, Kieszkowska-Grudny et al. [25] confirmed that the quality of life of patients with hip joint disease improved significantly in physical, emotional, and social aspects after hip arthroplasty. The authors emphasized a change in coping strategies from maladaptive to adaptive, which resulted in the subjects’ independence and lack of dependence on caregivers. Bodys-Cupak et al. [16] emphasized that the important factors in increasing quality of life included improved physical fitness, weight loss, increased acceptance of the disease, increased independence, and the possibility of self-care. This was also confirmed by our research. Mariconda et al. [26] proved that the obtained result is not short-term, and good quality of life is maintained in the long term, which was confirmed by their 16 years of observation. The increase in the quality of life in patients after hip arthroplasty was also confirmed in studies by Mandzuk et al. [27]. However, other studies confirmed that the level of postoperative satisfaction is high, but satisfaction with the quality of life in the physical aspect and functionality of the hip joint, in many cases, requires a longer time due to the process of achieving full independence [28,29,30,31,32].

Our research proves that modern hip arthroplasty improves health and improves the quality of life of patients. Eliminating pain, restoring mobility through early activation, and rehabilitation after the procedure contribute to a quick recovery and a satisfactory therapeutic effect for both the practitioner and the treated person.

The study has some limitations. Primarily, it was limited to one treatment center, and the study group was not very large because the study was conducted during the COVID-19 pandemic, during which, the number of arthroplasties performed decreased. In their study, Moldovan et al. [33] confirmed that the COVID-19 pandemic seriously affected the number of arthroplasties in hospitals in Romania, which also produced unfavorable financial consequences. The authors proposed a solution in the form of developing new procedures and alternative clinical solutions, as well as personalized home recovery programs, that will be activated, if necessary, if future pandemics occur.

## 6. Conclusions

Early rehabilitation after hip replacement surgery contributes to eliminating the disability of the hip joint in the following areas: pain sensation, functionality, lack of deformation, and range of motion. Each subsequent stage of treatment increased the level of acceptance of the disease in the study group. The surgical treatment led to an increased satisfaction with health and quality of life. The higher the level of acceptance of the disease, the higher the patient’s assessment of their health condition and quality of life in the social field.

## Figures and Tables

**Table 1:** Sociodemographic characteristics of the studied patients.

Sociodemographic Data		*N*	%
Gender	Woman	95	64.63%
	Man	52	35.37%
Age	≤50 years old	11	7.48%
	51–65 years old	52	35.37%
	66–80 years old	63	42.86%
	>80 years old	21	14.29%
Marital status	Married	104	70.75%
	Widower	38	25.85%
	Single	1	0.68%
	Divorced	4	2.72%
	Separated	0	0.00%
Place of residence	Village	69	46.94%
	Town	78	53.06%
Living and housing	Alone	13	8.84%
	Only with spouse	68	46.26%
	Spouse and children	38	25.85%
	Only with children	18	12.45%
	Other	10	6.80%
Education	Higher	5	3.40%
	Secondary	74	50.34%
	Vocational	68	46.26%
Living and housing conditions	Good	122	82.99%
	Average	25	17.01%
	Bad	-	-
	Very bad	-	-

**Table 2:** Pain assessment at each stage of treatment in the studied group of patients (VAS).

Parameter	STAGE I Before the Procedure	STAGE II 12 Days after the Procedure	STAGE III 6 Weeks after the Procedure
Mean ± SD	4.79 ± 1.25	4.69 ± 0.65	1.55 ± 0.82
(CI 95%)	(4.59–4.99)	(4.59–4.80)	(1.42–1.68)
Median	5	5	1
Min–max	3–7	4–6	1–4

**Table 3:** Distribution of HHS scale results in the studied group of patients.

Parameter	STAGE I Before the Procedure	STAGE II 12 Days after the Procedure	STAGE III 6 Weeks after the Procedure
Mean ± SD	67.60 ± 35.91	54.84 ± 7.14	80.12 ± 4.04
(CI 95%)	61.74–73.45	53.68–56.00	79.46–80.77
Median	85	56	81
Min–max	0–95	30–65	71–90

**Table 4:** Distribution of Barthel Index results in the studied group of patients.

Parameter	STAGE I Before the Procedure	STAGE II 12 Days after the Procedure	STAGE III 6 Weeks after the Procedure
Mean ± SD	83.40 ± 23.60	90.54 ± 2.93	96.97 ± 3.58
(CI 95%)	(79.55–87.25)	(90.07–91.02)	(96.08–97.25)
Median	95	90	95
Min–max	30–100	80–95	85–100
Profound disability	0.00%	0.00%	0.00%
Severe disability	21.77%	0.00%	0.00%
Moderate disability	0.00%	0.00%	0.00%
Mild disability	57.82%	100%	53.74%
No disability	20.41%	0.00%	46.26%

**Table 5:** Distribution of AIS results in the studied group of patients.

Parameter	STAGE I Before the Procedure	STAGE II 12 Days after the Procedure	STAGE III 6 Weeks after the Procedure
Mean ± SD	28.68 ± 5.02	29.81 ± 3.68	36.50 ± 2.01
(CI 95%)	(27.86–29.50)	(29.21–30.41)	(36.17–36.82)
Median	31	31	37
Min–max	17–37	20–34	30–38
AIS, low level (%)	8.84%	0.00%	0.00%
AIS, average level (%)	27.21%	31.29%	4.76%
AIS, high level (%)	63.95%	68.71%	95.24%

**Table 6:** Mean values of the WHOQOL-BREF score in individual domains.

WHOQOL-BREF Domains	STAGE I Before the Procedure Mean ± SD (Cl-95%)	STAGE II 12 Days after the Procedure Mean ± SD (Cl-95%)	STAGE III 6 Weeks after the Procedure Mean ± SD (Cl-95%)
Physical	14.65 ± 2.21 (14.29–15.01)	14.29 ± 2.24 (13.93–14.65)	16.36 ± 1.77 (16.07–16.65)
Psychological	13.19 ± 2.04 (12.86–13.52)	12.63 ± 1.84 (12.33–12.93)	13.69 ± 1.98 (13.37–14.01)
Social	14.39 ± 2.26 (14.02–14.76)	14.10 ± 2.32 (13.72–14.48)	14.36 ± 2.08 (14.02–14.70)
Environmental	14.12 ± 1.69 (13.85–14.39)	13.79 ± 1.72 (13.51–14.07)	14.39 ± 1.51 (14.15–14.63)

**Table 7:** The impact of acceptance of illness (AIS) on overall quality of life and in specific areas (WHOQOL-BREF).

AIS	STAGE I Before the Procedure Mean ± SD (CI 95%)	STAGE II 12 Days after the Procedure Mean ± SD (CI 95%)	STAGE III 6 Weeks after the Procedure Mean ± SD (CI 95%)
General quality of life			
Low level	3.00 ± 0.00 (3.0–3.0)	-	-
Average level	3.20 ± 0.41 (3.13–3.27)	3.19 ± 0.40 (3.13–3.25)	3.00 ± 0.00 (3.0–3.0)
High level	3.82 ± 0.41 (3.75–3.89)	3.76 ±0.45 (3.70–3.82)	3.73 ± 0.50 (3.65–3.81)
ANOVA test Kruskal–Wallis test	H = 61.002 *p* < 0.001	H = 39.960 *p* = <0.001	H = 20.215 *p* = 0.0002
Physical domain			
Low level	10.69 ± 0.63 (10.59–10.79)	-	-
Average level	13.27 ± 1.32 (13.06–13.48)	12.39 ± 1.50 (12.15–12.63)	13.28 ± 1.25 (13.08–13.48)
High level	15.78 ± 1.61 (15.52–16.04)	15.16 ±1.98 (14.84–15.48)	16.51 ± 1.65 (16.24–16.78)
ANOVA test Kruskal–Wallis test	H = 87.258 *p* < 0.001	H = 56.804 *p* < 0.001	H = 51.830 *p* < 0.001
Psychological domain			
Low level	9.69 ± 0.48 (9.61–9.77)	-	-
Average level	11.62 ± 1.23 (11.42–11.82)	10.74 ± 1.81 (10.45–11.03)	10.28 ± 0.49 (10.20–10.36)
High level	14.34 ± 1.33 (14.12–14.56)	13.49 ±1.38 (13.27–13.71)	13.86 ± 1.87 (13.56–14.16)
ANOVA test Kruskal–Wallis	H = 106.235 *p* < 0.001	H = 86.540 *p* < 0.001	H = 106.580 *p* < 0.001
Social domain			
**Parameter**	**STAGE I** **Before the Procedure**	**STAGE II** **12 Days after the Procedure**	**STAGE III** **6 Weeks after the Procedure**
Low level	12.23 ± 1.92 (11.92–12.54)	-	-
Average level	12.55 ± 2.04 (12.22–12.88)	11.69 ± 1.30 (11.48–11.90)	11.00 ± 0.00 (11.00–11.00)
High level	15.47 ± 1.59 (15.21–15.73)	15.20 ± 1.80 (14.91–15.49)	14.53 ± 1.99 (14.21–14.85)
ANOVA test Kruskal–Wallis	H = 72.976 *p* < 0.001	H = 85.799 *p* < 0.001	H = 59.768 *p* < 0.001
Environmental domain			
Low level	11.23 ± 1.01 (11.07–11.39)	-	-
Average level	13.05 ± 0.84 (12.91–13.19)	12.19 ± 1.42 (11.96–12.42)	11.86 ± 1.46 (11.62–12.10)
High level	14.98 ± 1.28 (14.77–15.19)	14.51 ± 1.31 (14.30–14.72)	14.52 ± 1.40 (14.29–14.75)
ANOVA test Kruskal–Wallis	H = 97.308 *p* < 0.001	H = 68.911 *p* < 0.001	H = 64.382 *p* < 0.001

**Table 8:** Spearman rank correlation between the level of pain experienced (VAS) and the quality of life of patients (WHOQOL-BREF).

WHOQOL-BREF	STAGE I Before the Procedure	STAGE II 12 Days after the Procedure	STAGE III 6 Weeks after the Procedure
General quality of life	−0.2978 *	−0.3679 *	−0.5462 *
Physical domain	−0.6810 *	−0.6586 *	−0.6451 *
Psychological domain	−0.6694 *	−0.5492 *	−0.6903 *
Social domain	−0.4929 *	−0.6668 *	−0.5775 *
Environmental domain	−0.6086 *	−0.4824 *	−0.7090 *

* The strength of correlation: |rs| < 0.3, no correlation; 0.3 ≤ |rs| < 0.5, weak correlation; 0.5 ≤ |rs| < 0.7, average correlation; 0.7 ≤ |rs| < 0.9, strong correlation; 0.9 ≤ |rs| ≤ 1, very strong correlation.

**Table 9:** Spearman rank correlation coefficients between psychometric measures.

Scale	HHS	Barthel	VAS	AIS	Quality of Life	Quality of Health	Physical Domain	Psychological Domain	Social Domain	Environmental Domain
Stage I of treatment										
HHS	1	0.679 *	0.735 *	0.842 *	0.686 *	0.808 *	0.781 *	0.822 *	0.669 *	0.744 *
Barthel	0.679 *	1	0.848 *	0.840 *	0.188 *	0.500 *	0.369 *	0.520 *	0.321 *	0.415 *
VAS	0.735 *	0.848 *	1	0.845 *	0.280 *	0.512 *	0.603 *	0.637 *	0.596 *	0.571 *
AIS	0.842 *	0.840 *	0.845 *	1	0.447 *	0.584 *	0.546 *	0.580 *	0.487 *	0.497 *
Quality of life	0.686 *	0.188 *	0.280 *	0.447 *	1	0.520 *	0.690 *	0.713 *	0.613 *	0.774 *
Quality of health	0.808 *	0.500 *	0.512 *	0.584 *	0.520 *	1	0.682 *	0.731 *	0.351 *	0.628 *
Physical domain	0.781 *	0.369 *	0.603 *	0.546 *	0.690 *	0.682 *	1	0.797 *	0.658 *	0.824 *
Psychological domain	0.822 *	0.520 *	0.637 *	0.580 *	0.713 *	0.731 *	0.797 *	1	0.726 *	0.875 *
Social domain	0.669 *	0.321 *	0.596 *	0.487 *	0.613 *	0.351 *	0.658 *	0.726 *	1	0.753 *
Environmental domain	0.744 *	0.415 *	0.571 *	0.497 *	0.774 *	0.628 *	0.824 *	0.875 *	0.753 *	1
Stage II of treatment										
HHS	1	0.112	0.399 *	0.615 *	0.732 *	0.673 *	0.548 *	0.681 *	0.719 *	0.671 *
Barthel	0.112	1	0.545 *	0.636 *	0.042	0.005	0.293 *	0.218 *	0.270 *	0.008
VAS	0.399 *	0.545 *	1	0.695 *	0.346 *	0.516 *	0.661 *	0.552 *	0.650 *	0.415 *
AIS	0.615 *	0.636 *	0.695 *	1	0.364 *	0.396 *	0.603 *	0.597 *	0.647 *	0.388 *
Quality of life	0.732 *	0.042	0.346 *	0.364 *	1	0.584 *	0.444 *	0.730 *	0.662 *	0.747 *
Quality of health	0.673 *	0.005	0.516 *	0.396 *	0.584 *	1	0.465 *	0.488 *	0.515 *	0.436 *
Physical domain	0.548 *	0.293 *	0.661 *	0.603 *	0.444 *	0.465 *	1	0.815 *	0.781 *	0.616 *
Psychological domain	0.681 *	0.218 *	0.552 *	0.597 *	0.730 *	0.488 *	0.815 *	1	0.903 *	0.840 *
Social domain	0.719 *	0.270 *	0.650 *	0.647 *	0.662 *	0.515 *	0.781 *	0.903 *	1	0.827 *
Environmental domain	0.671 *	0.008	0.415 *	0.388 *	0.747 *	0.436 *	0.616 *	0.840 *	0.827 *	1
Stage III of treatment										
HHS	1	0.211 *	0.547 *	0.588 *	0.673 *	0.631 *	0.544 *	0.694 *	0.7148	0.631 *
Barthel	0.211 *	1	0.138 *	0.581 *	0.522 *	0.313 *	0.344 *	0.385 *	0.362 *	0.269 *
VAS	0.547 *	0.138 *	1	0.461 *	0.439 *	0.679 *	0.525 *	0.613 *	0.573 *	0.744 *
AIS	0.588 *	0.581 *	0.461 *	1	0.633 *	0.271 *	0.620 *	0.614 *	0.567 *	0.427 *
Quality of life	0.673 *	0.522 *	0.439 *	0.633 *	1	0.518 *	0.614 *	0.718 *	0.737 *	0.662 *
Quality of health	0.631 *	0.313 *	0.679 *	0.271 *	0.518 *	1	0.320 *	0.513 *	0.552 *	0.720 *
Physical domain	0.544 *	0.344 *	0.525 *	0.620 *	0.614 *	0.320 *	1	0.697 *	0.683 *	0.600 *
Psychological domain	0.694 *	0.385 *	0.613 *	0.614 *	0.718 *	0.513 *	0.697 *	1	0.938 *	0.826 *
Social domain	0.714 *	0.362 *	0.573 *	0.567 *	0.737 *	0.552 *	0.683 *	0.938 *	1	0.831 *
Environmental domain	0.631 *	0.269 *	0.744 *	0.427 *	0.662 *	0.720 *	0.600 *	0.826 *	0.831 *	1

* The strength of correlation: |rs| < 0.3, no correlation; 0.3 ≤ |rs| < 0.5, weak correlation; 0.5 ≤ |rs| < 0.7, average correlation; 0.7 ≤ |rs| < 0.9, strong correlation; 0.9 ≤ |rs| ≤ 1, very strong correlation.

## Data Availability

Data will be made available upon request to the corresponding author.
